# Relative risk of keloids in patients with chronic inflammatory skin disorders

**DOI:** 10.1016/j.jdin.2025.01.006

**Published:** 2025-01-28

**Authors:** Raphaella Lambert, Hannah Verma, Grace Rabinowitz, Nicholas Gulati, Jonas A. Adalsteinsson

**Affiliations:** Kimberly and Eric J. Waldman Department of Dermatology, Icahn School of Medicine at Mount Sinai, New York, New York

**Keywords:** atopic dermatitis, epidemiology, hidradenitis suppurativa, keloid, psoriasis, scar, wound healing

*To the Editor:* Keloids are an aberrant scar formation associated with chronic inflammation.^1^^,^^2^ Research suggests that patients with atopic dermatitis (AD) are at increased risk of developing keloids^3^^,^^4^; less is known about such risk among patients with other inflammatory skin diseases (ISDs), including hidradenitis suppurativa (HS) or psoriasis.^1^ Patients with ISDs often experience pruritus, resulting in scratching, skin damage and, potentially, scarring. These individuals may also be subject to invasive procedures, including incision and drainage or excision, which may contribute to keloid formation. Given the importance of understanding the broader implications of keloid formation in ISDs, we investigated if patients with HS, AD, and psoriasis are at increased risk of developing keloids as compared to each other and to healthy controls.

We conducted a retrospective, cross-sectional study utilizing the Mount Sinai Health System TriNetX network. Three patient cohorts were identified using International Classification of Diseases, Tenth Revision (ICD-10) codes: L73.2 (HS), L20 (AD), and L40 (psoriasis). Adult patients (18 years or older) were included for analysis, comprising 5170 with HS, 31,590 with AD, and 29,330 with psoriasis. Cohorts were compared to each other and to healthy controls (adult patients without HS, AD, or psoriasis) and 1:1 propensity score matched for age, sex, race, and a history of surgical procedures that may contribute to keloid development, including incision and drainage or excision. The relative risk for diagnosis of keloid was evaluated for each cohort.

Patients with HS, AD, and psoriasis demonstrated a significantly higher risk of keloids than healthy controls (relative risk: 2.365, 95% confidence interval: 1.545-3.622; 1.945, 1.574-2.402; 1.424, 1.128-1.798, respectively) (Table I). HS patients had a significantly higher risk of keloids than psoriasis patients (2.018, 1.304-3.122); however, compared to AD patients, there was no significant difference (1.167, 0.828-1.643) (Table I). AD patients also demonstrated a significantly higher risk of keloids compared to psoriasis patients (1.463, 1.148-1.863) (Table I).

**Table I:** Relative risk of keloid diagnosis among patients with HS, AD, and psoriasis as compared to each other and to healthy controls

Cohort	Healthy controls	95% CI	AD	95% CI	HS	95% CI	Psoriasis	95% CI
AD	1.945∗	1.574-2.402	-	-	-	-	1.463∗	1.148-1.863
HS	2.365∗	1.545-3.622	1.167	.828-1.643	-	-	2.018∗	1.304-3.122
Psoriasis	1.424∗	1.128-1.789	-	-	-	-	-	-

*AD*, Atopic dermatitis; *HS*, hidradenitis suppurativa.

∗ Denotes a statistically significant difference.

Keloids are a debilitating form of pathologic wound healing associated with high morbidity and bothersome symptoms, including pain and pruritus.^1^ We demonstrate that patients with certain ISDs have an elevated risk of keloid diagnosis compared to healthy individuals. Notably, HS and AD patients may face a higher risk of keloid diagnosis than psoriasis patients. Keloids and AD may share common molecular pathways, including those linked to the ADAM33-related gene and T helper (Th) 1, 2, and 17 cell functions.^1^^,^^3^ Research has also noted a shared role for Th2 and Th17 signaling pathways in both HS and AD; however, the precise nature of this association is unclear.^5^

Increased risk of keloids among patients with ISDs carries significant clinical implications. As severe HS may require invasive surgical interventions, such as sinus tract excisions or incision and drainage, dermatologists should be mindful of the heightened risk of keloids in this population, which may influence management. Limitations of this work include its single-center, retrospective design and reliance on pairwise associations. Additionally, using TriNetX, we cannot match for certain important confounders or mediators, such as the number of dermatologist visits per patient. Further research into keloid pathogenesis among patients with ISDs is warranted.

## Conflicts of interest

None disclosed.
